# Dopamine-dependent visual attention preference to social stimuli in nonhuman primates

**DOI:** 10.1007/s00213-017-4544-6

**Published:** 2017-02-02

**Authors:** Yoshie Yamaguchi, Takeshi Atsumi, Romain Poirot, Young-A Lee, Akemi Kato, Yukiori Goto

**Affiliations:** 1 Primate Research Institute, Kyoto University, 41-2 Kanrin, Inuyama, Aichi 484-8506 Japan; 2 Department of Rehabilitation for Brain Functions, Research Institute of Rehabilitation Center for Persons with Disabilities, Tokorozawa, Saitama, 359-8555 Japan; 3 Ecole Nationale Veterinaire de Toulouse, 31076 Toulouse, France; 4 Department of Food Science and Nutrition, Catholic University of Daegu, Gyeongsan, Gyounbuk 38430 South Korea

**Keywords:** Social cognition, Social reward, Macaque, Visual attention, Dopamine, Psychiatric disorder

## Abstract

**Rationale:**

Dopamine (DA) plays a central role in reward processing. Accumulating evidence suggests that social interaction and social stimuli have rewarding properties that activate the DA reward circuits. However, few studies have attempted to investigate how DA is involved in the processing of social stimuli.

**Objectives:**

In this study, we investigated the effects of pharmacological manipulations of DA D1 and D2 receptors on social vs. nonsocial visual attention preference in macaques.

**Methods:**

Japanese macaques were subjected to behavioral tests in which visual attention toward social (monkey faces with and without affective expressions) and nonsocial stimuli was examined, with D1 and D2 antagonist administration.

**Results:**

The macaques exhibited significantly longer durations of gazing toward the images with social cues than did those with nonsocial cues. Both D1 and D2 antagonist administration decreased duration of gazing toward the social images with and without affective valences. In addition, although D1 antagonist administration increased the duration of gazing toward the nonsocial images, D2 antagonism had no effect.

**Conclusions:**

These results suggest that both D1 and D2 receptors may have roles in the processing of social signals but through separate mechanisms.

## Introduction

Since the pioneering work by Olds and Milner (Olds and Milner 1954), the mesolimbic dopamine (DA) pathway, consisting of the ventral tegmental area (VTA), and DA neuron projections into the ventral striatum, have been demonstrated to play a critical role in reward processing (Phillips et al. 2008; Schultz 2013). In addition, extensive studies have revealed that mesocortical DA innervations in the prefrontal cortex (PFC) is also involved in multiple facets of cognitive and affective functions (Goldman-Rakic et al. 2000; Klanker et al. 2013; Robbins 2000; Salgado-Pineda et al. 2005). In addition to cognitive and affective functions, emerging evidence suggests that DA is also significantly involved in social functions (Skuse and Gallagher 2009). For instance, both D1 and D2 receptor antagonists have been shown to attenuate social interactions between mates in rodents (Corbett et al. 1993). Additionally, optogenetic stimulation of D1 receptors in the ventral striatum has been demonstrated to promote social interactions in mice (Gunaydin et al. 2014). In contrast, in pair bond formations in prairie voles, D1 receptor activation is found to prevent pair bonding, whereas D2 receptor activation facilitates pair bonding (Gingrich et al. 2000). A possible explanation for DA involvement in the regulation of social behaviors is the likely association with the rewarding properties of social stimuli, which activate the DA reward circuits (Krach et al. 2010; Trezza et al. 2011). However, studies have also demonstrated that DA release is promoted not only by affiliative (positive) social interactions but also by aggressive (negative) social interactions (Louilot et al. 1986). The roles of DA in the social functions of primates are less clear. In humans, negative and positive correlations of striatal D2 and D1 receptor availability, respectively, have been observed with social desirability (Cervenka et al. 2010; Plaven-Sigray et al. 2014); although in this case, the causal relationship remains unclear.

To elucidate how the DA system may be involved in social functions, in this study, we investigated the effects of pharmacological manipulations of DA D1 and D2 receptors on visual attention to social and nonsocial stimuli in nonhuman primates. Previous studies have shown that DA has important roles in visual attention in rodents (Granon et al. 2000), nonhuman primates (Noudoost and Moore 2011), and human subjects (Muller et al. 1998). However, the visual stimuli used for attention tests in these studies were independent of ecological contexts, such as flashing lights or simple shape figures (e.g., dots, squares). We conducted a visual preference test in which macaques were exposed to images of objects (nonsocial cues) and monkey faces with and without emotional expressions (social cues with/without affective valences) in conditions of either D1 or D2 receptor antagonist administration. We hypothesized that macaques exhibited preferential attention toward images with social cues rather than nonsocial images. Moreover, this social preference might be disrupted by D1 and D2 receptor blockades.

## Materials and methods

### Subjects and drug administration

All experiments were conducted in accordance with the *Science Council of Japan Guidelines for Proper Conduct of Animal Experiments* and were approved by the Kyoto University Primate Research Institute Animal Experiment Committee. Two 5-year-old male Japanese macaques (M1, M2) were used in this study. These monkeys were housed individually with food and water freely available throughout the experiments.

The D1 antagonist SCH23390 (SCH) was dissolved in 3.0 ml of 0.9% saline and was given to the subjects at a dose of 0.5 mg/kg (i.m.), which is a relatively high dose compared to doses used in other studies (Arnsten et al. 1994; Von Huben et al. 2006). We selected this dose, because we found that drug administration at a lower dose (0.1 mg/kg) did not cause a clear impairment in separate memory test in these macaques (data not shown). The D2 antagonist sulpiride (SUL) was dissolved in a drop of 1 N HCl and diluted with 0.9% saline for a final volume of 6.0 ml, which was given to the subjects subcutaneously at the dose of either 4.5 mg/kg (low dose; l-SUL) or 45 mg/kg (high dose; h-SUL). We examined these two doses of the D2 antagonist because we noticed that administration of the high dose, but not low dose, of SUL caused substantial motor effects such as stereotypy, which might have potentially interfered with task engagement of the subjects. An equivalent volume of saline (SAL) was given as a control in both SCH and SUL administrations. Drug administration was conducted approximately 3 h before the behavioral test.

### Visual attention preference tests

**Fig. 1:**
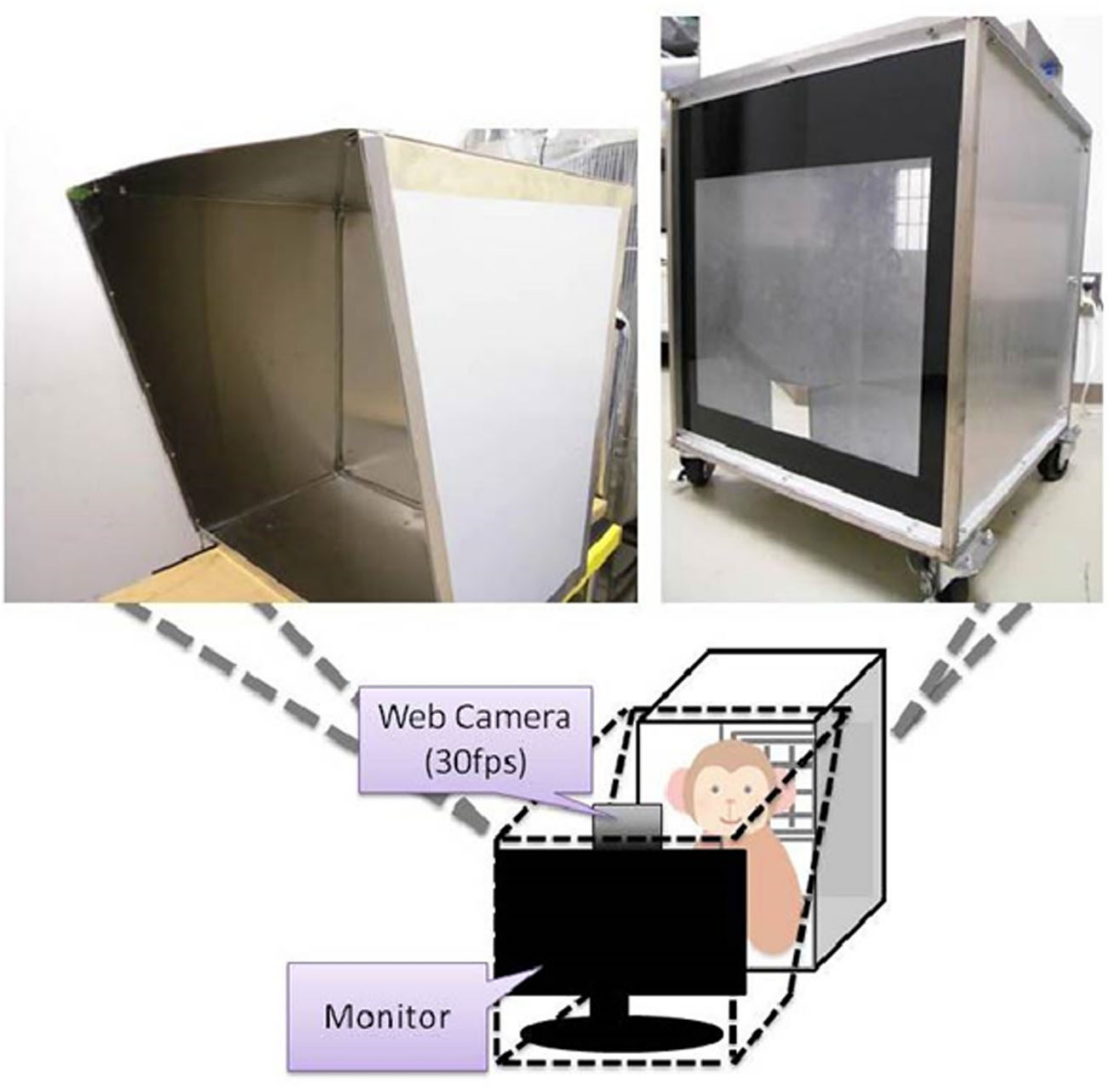
Visual attention preference test in nonhuman primates. A diagram illustrating the custom-made setup used for the test

The visual attention preference test was conducted using a custom-made operant box (Fig. 1). The 14″ LCD screen was attached to the operant box and images were presented on the screen. The images were divided into three categories. One category was nonsocial images (NS), such as trees, animals other than primates, foods (e.g., potato, apple), flowers, and landscapes. The other two categories were social images, both of which were macaque faces, but one category was without emotional expressions (neutral faces; SNT), and the other category was with threatening facial expressions showing teeth (emotional faces; SEM). All images were obtained from the internet (and thereby copy-protected) and adjusted to approximately equal sizes. Before presenting each image, a brief sound was emitted to attract attention to the screen. When the subjects oriented their attention toward the screen, an image was presented. Spontaneous gazes toward the screen during image presentation were monitored and video-recorded with the video camera on the top of the LCD screen. Duration and frequency of gazing upon the images were analyzed later off-line using the software Adobe Premiere Pro CC 2015.

The visual attention preference tests were conducted in two different conditions: (1) single image test and (2) paired image test. First, we examined the single image test condition in which a single image was presented on the LCD screen in each trial. In this test condition, each category of NS, SNT, and SEM had 10 different images for a total of 30 images presented in one session. Each image was presented for 5 s. Intertrial intervals were arbitrarily set at 5∼30 s. Five sessions were given to each subject, with drug administration in the following order; SAL (SAL1)-- > SCH-- > SAL (SAL2)-- > h-SUL-- > l-SUL. Each session was executed with intervals of 5∼7 days between the sessions. Upon completion of the single image test condition, we further examined the paired image test condition in which a pair of images, with a combination of either NS-SNT, NS-SEM, or SNT-SEM, was presented on the left and the right side of the LCD screen, respectively, in a counter-balanced manner in each trial. Each pair of NS-SNT, NS-SEM, and SNT-SEM had 10 different patterns for a total of 30 pairs of images presented in one session. Each pair of images was presented for 10 s. Five sessions were given again to each subject, with the same drug administration order as given in the single image test condition.

### Data analysis

Data collection and statistical analyses were conducted by investigators who were not blinded to the experimental conditions. No data points were removed from statistical analysis. Sample sizes were not predetermined by statistical methods. Analysis of variance (ANOVA) was conducted with Bonferroni correction (post hoc Bonferroni test). All statistical analyses were conducted using Statistica software. A probability value of *p* < 0.05 was considered to indicate statistical significance.

## Results

### Single image test

Attention preference toward social vs. nonsocial images was first examined in the condition of single image presentation in each trial.

**Fig. 2:**
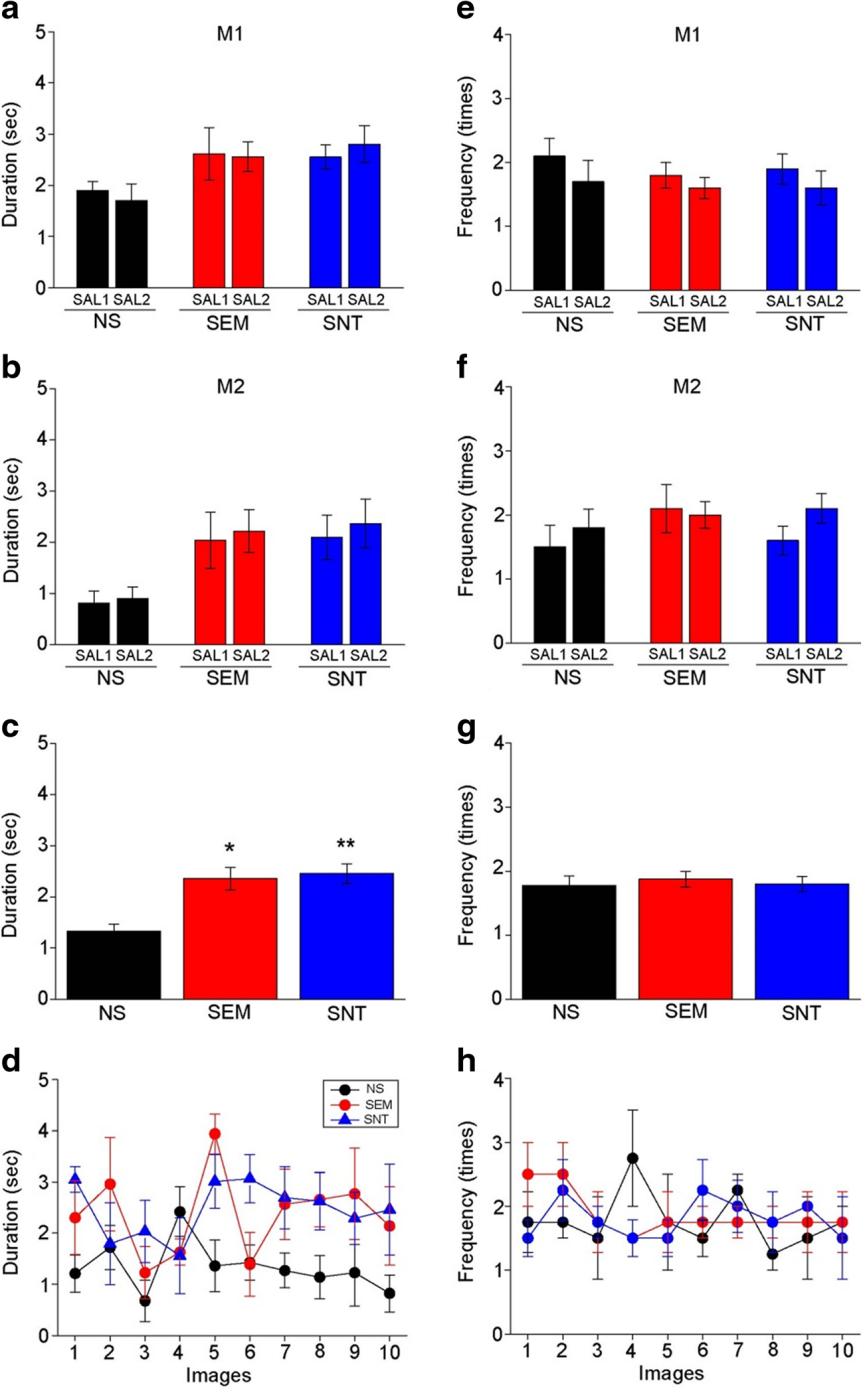
Duration and frequency of gazing on the social and nonsocial images in the single image test with saline administration. **a**, **b** Graphs showing duration of gazing separately for each subject (**a** M1, **b** M2) toward the nonsocial images (*NS*) and social images with (*SEM*) and without (*SNT*) affective valences with two SAL administrations (*SAL1*, *SAL2*). *Error bars* indicate s.e.m. **c** A graph showing duration of gazing toward the images with the data shown in **a**, **b** combined. *p < 0.001; **p < 0.001, with post hoc Bonferroni test. **d** A graph showing duration of gazing toward 10 single images in each category of NS, SEM, and SNT. **e**–**h** Graphs similar to **a**–**d**, but instead showing frequency of gaze orientations toward the images

The macaques M1 and M2 exhibited a similar pattern of gaze duration toward nonsocial images and social images with and without affective valences with control SAL treatments (M1, two-way ANOVA, *F*_1,54_ = 0.290, *p* = 0.592 for treatments [SAL1 vs. SAL2]; *F*_2,54_ = 7.105, *p* = 0.002 for categories [NS vs. SEM vs. SNT]; *F*_2,54_ = 0.024, *p* = 0.976 for interaction [treatments × categories]; M2, *F*_1,54_ = 0.0001, *p* = 0.994 for treatments; *F*_2,54_ = 4.191, *p* = 0.020 for categories; *F*_2,54_ = 0.236, *p* = 0.791 for interaction; Fig. 2a, b). Since there was no statistically significant difference between SAL1 and SAL2 or between M1 and M2, these data were combined in the subsequent analysis. A one-way ANOVA revealed that the duration of gazing toward social images was significantly longer than that toward nonsocial images (*F*_2,117_ = 11.16, *p* < 0.001; Fig. 2c). However, there was no difference between social images with and without affective valences (Fig. 2c). No particular image elicited a duration of gazing with SAL condition that was substantially deviated from other images in any category (Fig. 2d). Frequency to orient gaze toward an image in each trial was also measured in the SAL condition. In contrast to gaze duration, no significant difference between categories was observed (Fig. 2e–h).

**Fig. 3:**
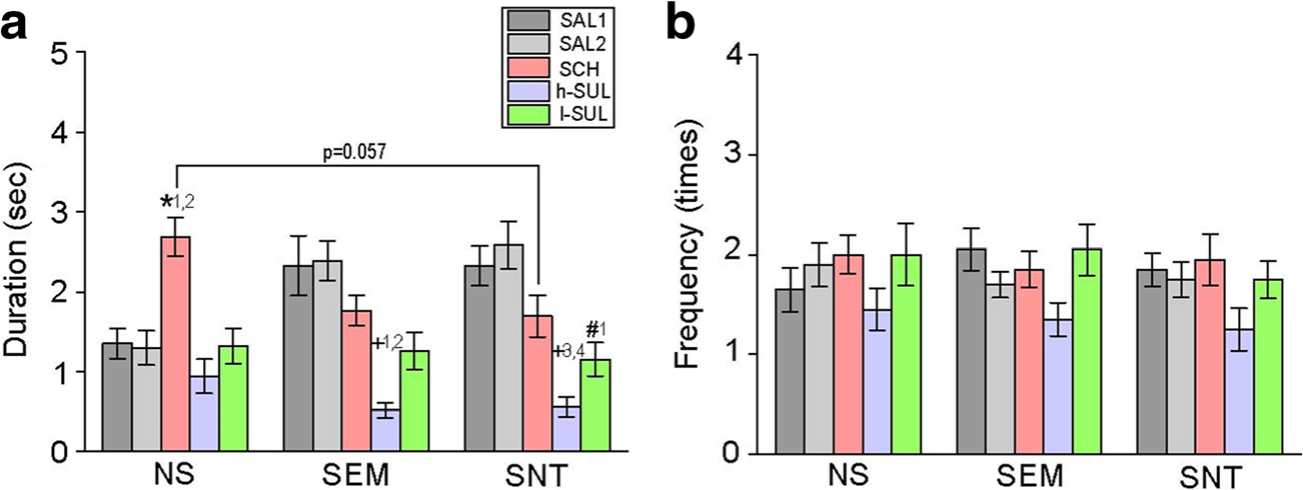
Effects of the DA antagonists in the single image test. **a** A graph showing duration of gazing toward the images with saline (*SAL1*, *SAL2*) and D1 (*SCH*) and two different doses of D2 (*h-SUL* and *l-SUL*) antagonist administration. *^1^ *p* = 0.013 vs. SAL1 in NS, *^2^ *p* = 0.007 vs. SAL2 in NS, ^†1^ *p* < 0.001 vs. SAL1 in SEM, ^†2^ *p* < 0.001 vs. SAL2 in SEM, ^†3^ *p* < 0.001 vs. SAL1 in SNT, ^†4^ *p* < 0.001 vs. SAL2 in SNT, ^#1^ *p* = 0.004 vs. SAL2 in SNT. **b** A graph similar to **a**, but instead showing frequency of gaze orientations toward the images

Next, alterations of gaze duration and frequency toward social and nonsocial images under SCH and SUL conditions were examined. A two-way ANOVA with repeated measures revealed significant effects of drug treatments and interactions between categories and treatments (*F*_2,57_ = 0.867, *p* = 0.426 for categories [NS vs. SEM vs. SNT]; *F*_4,228_ = 20.46, *p* < 0.001 for treatments [SAL1 vs. SAL2 vs. SCH vs. l-SUL vs. h-SUL]; *F*_8,228_ = 4.859, *p* < 0.001 for interaction [categories × treatments]; Fig. 3a). Gaze duration toward NS images was significantly increased under the SCH condition (Fig. 3a). In addition, although they did not reach statistical significance, gaze duration toward SEM and SNT images tended to be decreased, consequently resulting in marginally significant difference in duration between NS and SNT with SCH administration (Fig. 3a). In contrast, neither l-SUL nor h-SUL administration altered duration of gazing toward NS images, but duration toward SEM and SNT images was significantly decreased (Fig. 3a). Unlike gaze duration, although overall drug treatment effects were observed in frequency of orienting gazes toward the images (*F*_2,57_ = 0.387, *p* = 0.681 for categories; *F*_4,228_ = 3.732, *p* = 0.006 for treatments; *F*_8,228_ = 0.220, *p* = 0.987 for interaction; Fig. 3b), post hoc analysis found no significant difference in the comparison of each administration condition.

These results suggest that nonhuman primates exhibit a visual attention preference toward social over nonsocial signals. Moreover, both D1 and D2 receptors are involved in social preference, although their underlying mechanisms may be different.

### Paired image test

We further evaluated the results of the single image test condition by conducting another visual attention preference test with a different paradigm in which a pair of images with different categories was presented in each trial.

**Fig. 4:**
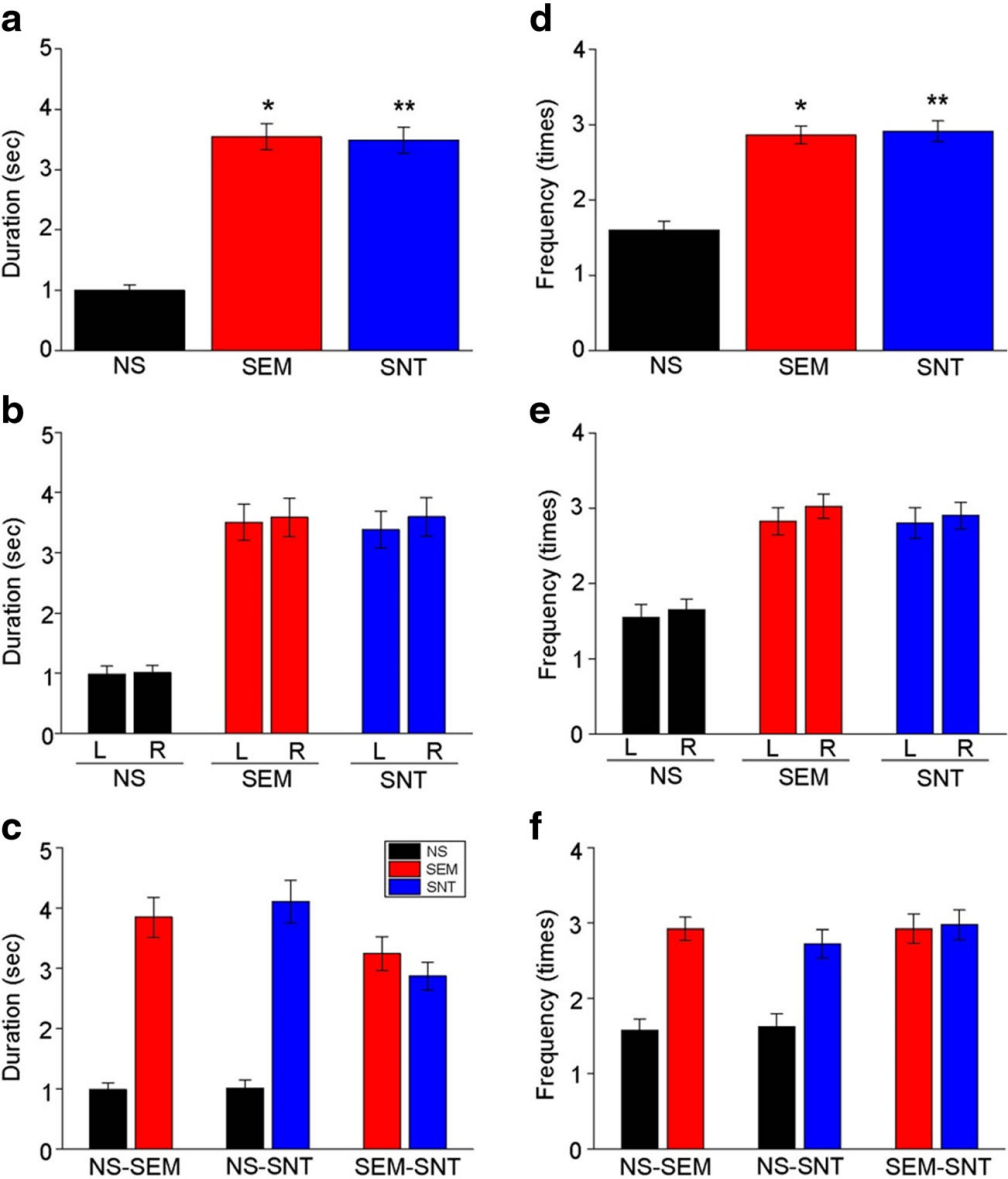
Duration and frequency of gazing toward the social and nonsocial images in the paired image test with saline administration. **a** A graph showing duration of gazing toward the nonsocial and social images with SAL administration. *p < 0.001; **p < 0.001. **b**, **c** Graphs showing duration of gazing toward the images with the data separately analyzed for the images presented on the *left* or the *right side* of the screen (**b**) and different combinations of the social and nonsocial images (**c**). **d**–**f** Graphs similar to **a**–**c**, but instead showing frequency of gaze orientations toward the images. *p < 0.001; **p < 0.001

Similar to the single image test condition, duration of gazing toward social images was significantly longer than that toward nonsocial images, although there was no difference between social images with and without affective valences in the SAL condition (*F*_2,237_ = 60.93, *p* < 0.001; Fig. 4a). The orientation of image presentation on the left or the right side of the screen (Fig. 4b), or a combination pattern of the images in each pair (Fig. 4c), was not associated with preferred gazing toward social over nonsocial images. Unlike the single image test condition, frequency of orienting gazes was significantly higher toward social images than that to nonsocial images in the paired image presentation condition (*F*_2,237_ = 36.35, *p* < 0.001; Fig. 4d). This difference in frequency between social and nonsocial images was also not associated with the left/right side presentation (Fig. 4e) or a combination pattern (Fig. 4f) in the pairs.

**Fig. 5:**
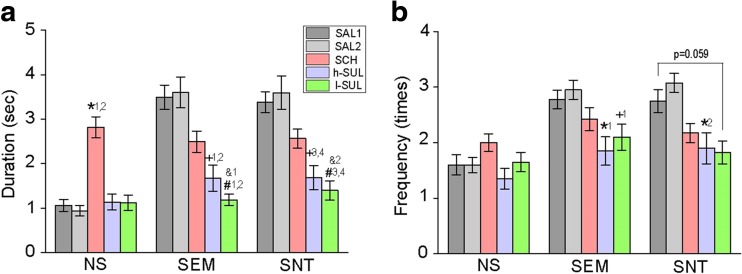
Effects of the DA antagonists in the paired image test. **a** A graph showing duration of gazing toward the images with SAL, SCH, l-SUL, and h-SUL administration. *^1^ *p* < 0.001 vs. SAL1 in NS, *^2^ *p* < 0.001 vs. SAL2 in NS, ^†1^ *p* < 0.001 vs. SAL1 in SEM, ^†2^ *p* < 0.001 vs. SAL2 in SEM, ^†3^ *p* < 0.001 vs. SAL1 in SNT, ^†4^ *p* < 0.001 vs. SAL2 in SNT, ^#1^ *p* < 0.001 vs. SAL1 in SEM, ^#2^ *p* < 0.001 vs. SAL2 in SEM, ^#3^ *p* < 0.001 vs. SAL1 in SNT, ^#4^ *p* < 0.001 vs. SAL2 in SNT, ^&1^ *p* = 0.038 vs. SCH in SEM, ^&2^ *p* = 0.036 vs. SCH in SNT. **b** A graph similar to **a**, but instead showing frequency of gaze orientations toward the images. *^1^ *p* = 0.022 vs. SAL1 in SEM, *^2^ *p* = 0.002 vs. SAL2 in SNT, ^†1^ *p* = 0.008 vs. SAL1 in SNT

SCH and SUL administration altered duration of gazing toward social and nonsocial images, which is consistent with observations in the single image test condition. SCH administration increased duration of gazing toward NS images (*F*_2,117_ = 39.92, *p* < 0.001 for categories; *F*_4,468_ = 26.0, *p* < 0.001 for treatments; *F*_8,468_ = 10.12, *p* < 0.001 for interaction; Fig. 5a). SCH administration, although not statistically significant in most, also tended to decrease duration of gazing toward SEM and SNT images (Fig. 5a). Neither l-SUL nor h-SUL administration altered duration of gazing toward NS images, but both l-SUL and h-SUL administration significantly decreased duration of gazing toward SEM and SNT images (Fig. 5a). Moreover, the decreases of gaze duration toward SEM and SNT images with l-SUL administration were greater than those with SCH administration (Fig. 5a). Frequency of gaze orientations toward social images was also decreased with SUL administration (*F*_2,117_ = 24.37, *p* < 0.001 for categories; *F*_4,468_ = 9.38, *p* < 0.001 for treatments; *F*_8,468_ = 2.35, *p* = 0.017 for interaction; Fig. 5b).

These results support the findings of the single image test demonstrating that macaques exhibit a visual attention preference toward social over nonsocial signals and that both D1 and D2 receptors may be involved in this social preference with different mechanisms.

## Discussion

DA has been shown to be involved in visual attention in rodents (Granon et al. 2000), nonhuman primates (Noudoost and Moore 2011), and human subjects (Muller et al. 1998). In these studies, visual cues for attention were ecologically independent ones. Our study has now demonstrated that ecological contexts, such as whether targets for attention are social or nonsocial, are important in DA-dependent visual attention. We found that nonhuman primates exhibit attention preferences for social over nonsocial cues. Moreover, DA mediates this social preference in visual attention through D1 and D2 receptors. However, no significant effect of the D1 and D2 antagonists was found in the processing of affective features embedded in social cues. Attention preference toward primate faces with and without emotional expressions was not different between control and drug administration conditions.

We examined the effects of both D1 and D2 receptor antagonist administration. In rodents, both D1 and D2 receptors have been shown to be involved in regulation of social interactions, although in one study, D1 and D2 receptors were shown to synergistically promote social interactions (Corbett et al. 1993), but another study showed that D1 and D2 receptor activation yielded the opposite effects on pair bonding (Gingrich et al. 2000). Thus, how D1 and D2 receptors work on the regulation of social behaviors could differ depending on the types of social activities. We found that the effects of visual attention preference toward social over nonsocial cues by the D1 and D2 antagonists were synergistic; however, the underlying mechanisms were different in nonhuman primates. Thus, D1 antagonist administration increased attention toward nonsocial images and slightly decreased attention toward social images, shifting the balance between social and nonsocial preference. In contrast, D2 antagonist administration substantially decreased attention toward social images but did not affect attention toward nonsocial images, suggesting that D2 receptor activation is selectively involved in producing social preference in visual attention. Studies have shown that social stimuli can be rewarding and therefore involve activation of the DA reward pathway (Krach et al. 2010; Trezza et al. 2011). The effects of the D2 antagonist may be explained by this rewarding process. On the other hand, the effects of the D1 antagonist may be explained by other mechanisms that are associated with cognitive processes. For instance, one of the suggested functions of the D1 receptor is to facilitate the signal-to-noise ratio of information processing (Seamans and Yang 2004), suggesting that D1 antagonist administration may promote more attention to less important (nonsocial) cues, and less attention to more important (social) cues.

Specific neural mechanisms associated with visual attention preference toward social cues observed in this study may be revealed in future studies. DA receptors are expressed in several brain areas including the prefrontal cortex (PFC) and striatum. DA in the PFC has been shown to play critical roles in visual attention (Granon et al. 2000; Noudoost and Moore 2011). Moreover, DA signaling in the nucleus accumbens (NAcc) mediates learned preference, including conditioned place preference toward social interactions (Trezza et al. 2011). Thus, attention preference toward social signals may involve coordinated DA-dependent PFC-NAcc information processing.

In this study, we examined visual attention with two different paradigms of image presentation. The first paradigm involved the presentation of a single image in each trial. The second paradigm involved the presentation of a pair of images with different categories in each trial. Preference toward social over nonsocial cues and the effects of DA modulation were essentially similar between these two test conditions. However, the frequency to orient gaze toward images was different between the testing conditions. In single image presentation, frequency was not different between social and nonsocial images, but in paired image presentation, frequency of gaze orientation toward social images was significantly higher than that toward nonsocial images. Although frequency was not affected by the DA antagonists in the single image test condition, DA antagonist administration altered the frequency in the paired image test condition. The reason for this difference remains unclear. However, these results indicate that some aspects of behaviors were influenced by the testing conditions.

In the processing of social cues, such as faces of macaque species, gender differences have been reported in nonhuman primates. For instance, in Sulawesi macaques, males give more attention to other macaque faces than females (Fujita and Watanabe 1995). Since we examined only male macaques in this study, therefore, it would be important to investigate whether attention preference toward social over nonsocial cues is similarly observed in female macaques through similar DA mechanisms. In addition, attention to other macaque faces has also been demonstrated to be influenced by the hierarchical rank of the subjects in social groups (Deaner et al. 2005; McNelis and Boatright-Horowitz 1998). However, this factor can be excluded in this study, because subjects were individually housed and the presented social images were of unfamiliar faces.

## Conclusions

Our study suggests that nonhuman primates exhibit a preference of visual attention toward social signals over nonsocial cues, regardless of the affective significance of the social cues. This process involves both DA D1 and D2 receptors, but through different mechanisms. Although D2 receptor activation selectively mediates social cue processing, D1 receptor activation balances both social and nonsocial cue processing.
